# Visual/anatomical outcome of diabetic macular edema patients lost to follow-up for more than 1 year

**DOI:** 10.1038/s41598-021-97644-2

**Published:** 2021-09-15

**Authors:** Ji Soo Kim, Seungheon Lee, Jin Young Kim, Eoi Jong Seo, Ju Byung Chae, Dong Yoon Kim

**Affiliations:** 1 Department of Ophthalmology, College of Medicine, Chungbuk National University Hospital, Chungbuk National University, 776, Sunhwan-1-Ro, Seowon-Gu, Cheongju, 28644 Korea; 2 Top Retina Center, 122, Gangseo-ro, Heungdeok-gu, Cheongju, 28378 Korea; 3 The One Seoul Eye Clinic, 624, Gangnam-daero, Gangnam-gu, Seoul, 06035 Korea

**Keywords:** Eye diseases, Retinal diseases

## Abstract

To investigate the visual/anatomical outcome of diabetic macular edema (DME) patients lost to follow-up (LTFU) for more than 1 year during intravitreal anti-VEGF treatment. A retrospective review of 182 treatment-naïve DME patients was performed. Among them, we identified patients LTFU for more than 1 year during anti-VEGF treatment. Visual acuity and anatomic outcomes at the first visit, last visit before being LTFU, return visit, and after re-treatment were analyzed and compared with those of DME patients with regular follow-up. Patients who had continuous follow-up visits were assigned to the control group. Sixty patients (33%) with DME were LTFU for more than 1 year during anti-VEGF treatment. Multivariate analysis revealed that the ratio of male (*p* = 0.004), diabetes mellitus (DM) duration less than 5 years (*p* = 0.015), and poor early anatomic response (*p* = 0.012) were higher compared to the control group. Eighteen patients returned to the clinic and received re-treatment. After re-treatment with anti-VEGF, central subfield thickness (CST) was significantly improved to the CST of before LTFU. However, visual acuity did not recover to the level before LTFU (0.63 ± 0.26 vs. 0.45 ± 0.28, *p* = 0.003). About thirty percent of DME patients were LTFU for more than 1 year. Permanent visual loss was observed in these LTFU patients. Patients with a high risk of LTFU such as male, early DM, and poor response after initial injections should be treated more aggressively to improve the visual outcomes.

## Introduction

Diabetic retinopathy (DR) is the most common cause of moderate and severe vision loss in working-age adults^1^. Diabetic macular edema (DME) is a major cause of vision loss in DR patients and is characterized by an accumulation of extracellular fluid in the macula due to increased vascular permeability^2^. With intravitreal anti-vascular endothelial growth factor (VEGF) and intravitreal dexamethasone implant treatment, the visual/anatomical prognosis of DME has improved^3–16^. However, the visual outcomes of DME patients in real-world clinical practice were relatively poorer than those in clinical trials^17,18^. Loss to follow-up (LTFU) during treatment might be one of the contributing factors that could lead to the poorer visual outcomes of DME patients in real-world practice, compared to those in clinical trials.

Maintaining treatment adherence in diabetic patients is important to improve the overall prognosis of diabetes and diabetes-related complications. However, up to 50% of diabetic patients are non-adherent to treatment^19,20^. The poor adherence to diabetic medication is strongly associated with poor glycemic control and a higher risk for micro-and macrovascular complications, while good adherence is associated with fewer emergency room and inpatient visits^20–22^. This poor adherence to treatment is related to several factors, such as higher disease and total medication burden, mood disorder, education level, and socioeconomic status^23–25^.

In the treatment of diabetic retinopathy, poor treatment adherence could lead to LTFU during treatment^26^. Recently, the prognosis of LTFU in proliferative diabetic retinopathy (PDR) patients following pan-retinal photocoagulation or intravitreal anti-VEGF injections has been reported^27,28^. In that study, the best-corrected visual acuity (BCVA) of PDR patients became significantly worse after the return visit from LTFU, regardless of the treatment method^27^.

Regarding treatment adherence in DME patients, about 28.8% of DME patients showed LTFU during anti-VEGF treatment in a previous report^29^. Among these LTFU patients, some returned and received re-treatment for DME. However, there is limited understanding of the visual/anatomical outcomes of re-treatment in LTFU DME patients. Therefore, we aimed to investigate the clinical outcome of DME patients who were lost to follow-up for more than 1 year during the anti-VEGF injection. We also tried to find characteristics of the LTFU patients during treatment.

## Methods

A retrospective review was conducted on treatment-naïve DME patients who had received bevacizumab injection at the Chungbuk National University Hospital, Cheongju, South Korea, between January 1, 2013, and December 31, 2017. The primary objective of this study was to analyze the visual/anatomical outcome of LTFU DME patients. The secondary objectives were to (1) know the rate of LTFU during anti-VEGF treatment and (2) determine the characteristics of those LTFU during treatment. All study participants provided informed consent for study participation and the publication of their data. This study was approved by the Institutional Review Board of the Chungbuk National University Hospital and followed the tenets of the Declaration of Helsinki.

The inclusion criteria were (1) treatment-naïve foveal involving DME with central subfield retinal thickness > 300 µm, (2) DME treatment with intravitreal bevacizumab injection, and (3) LTFU for more than 1 year after the last visit. Exclusion criteria included high myopia (> 8 diopters), glaucoma, media opacities due to cataract or corneal disease, vitreous hemorrhage, combined retinal disease, history of ocular trauma, or intraocular surgery, and poor-quality spectral-domain optical coherence tomography (SD-OCT) images. In the control group, we included treatment-naïve DME patients who had continuous follow-up visits with 1–3 months’ intervals.

### Ophthalmic examinations

At the initial visit, all patients underwent a comprehensive bilateral ophthalmic examination. This included BCVA using the Snellen chart, applanation tonometry, slit-lamp examination, fundus photography, and SD-OCT examination (Spectralis; Heidelberg Engineering, Heidelberg, Germany).

The BCVA results were converted to the LogMAR scale. At each visit, ophthalmic examinations including BCVA measurement, applanation tonometry, slit-lamp examination, dilated fundus examinations, fundus photography, and SD-OCT were performed.

### OCT examination and interpretation

The central subfield thickness (CST) was defined as the mean retinal thickness in a 1-mm diameter circular zone centered on the fovea and was automatically calculated. “Persistent DME” was defined as DME present after the first three consecutive anti-VEGF injections with CST ≥ 300 μm in SD-OCT^30^. When the patient has persistent DME at 12 weeks, we defined that as “poor early anatomic response”.

Also, the integrity of the ellipsoid zone was analyzed with horizontal and vertical radial scan within a radius of 1500 um centered on the fovea^7^. Integrity or discontinuity of the ellipsoid zone was evaluated differentiating between ‘defect present’ and ‘defect absent’^31^. Also, the lengths of ellipsoid zone defects were measured using the caliper function of the Spectralis® instrument. The mean of the values obtained in the horizontal and vertical scans was calculated. All OCT images were evaluated by two retinal specialists (C.J.B and K.J.Y).

### Treatment of diabetic macular edema

Bevacizumab was used as initial treatment in the treatment-naïve DME patients. All patients were treated with 3-monthly consecutive intravitreal bevacizumab injections (IVBI). Subsequently, bevacizumab was injected every 4 weeks until treatment responsiveness was achieved. Treatment response was defined as an increase in visual acuity of one or more Snellen lines (5 letter score) or a BCVA of 20/20, or a decrease in the CST by 10% or more, after three consecutive IVBIs^32^. If DME eyes achieved treatment response, IVBI was deferred to the next 4 weeks. After two consecutive treatment deferrals, the treatment interval was gradually extended. Then, we extended the treatment deferral interval up to 12 weeks. Subsequently, if there was no further worsening, we observed the DME patients at 12-week intervals.

### Patients lost to follow-up

Patients who had a history of LTFU for more than 1 year after the last visit were assigned to the LTFU group. DME patients who had a continuous follow-up visit with 1–3 months’ intervals were assigned to the control group. Also, patients who returned to our clinic within 12 months, even if they did not visit their scheduled appointment, were assigned to the control group^28^.

The characteristics of the LTFU group such as age, sex, distance from home to hospital, duration of diabetes mellitus, hemoglobin A1c (HbA1C), BCVA, and CST were compared to those of the control group.

Among the LTFU patients, some patients returned to the hospital, who we considered as a return group. If the returned patients had a foveal involving DME (CST > 300 µm), intravitreal bevacizumab re-injection was performed for DME treatment. To know the effects of LTFU on the visual/anatomical outcomes in DME patients, the BCVA and CST of the return group were investigated at the baseline, before LTFU, at return, and after re-treatment, and compared to the BCVA of the control group. And we also compared the ellipsoid zone change between the return group and the control group.

### Statistical analysis

All statistical tests were performed using SPSS, Version 24 (IBM Corp., Armonk, NY, USA). Shapiro–Wilk test was used to assess the normality. Independent t-tests were used to compare the results between the LTFU group and the control group. Differences in rates between categorical factors were assessed using a chi-square test. Paired t-tests were used to compare the results of the return group. Repeated measure ANOVA test was done to analyze the BCVA/CST change in the return group. Multivariate logistic regression analysis was used to determine the predictive factors for LTFU. Statistical significance was considered as a *p*-value of < 0.05.

### Consent to participate

As it is a retrospective study, IRB granted an informed consent waiver.

### Meeting presentations

Paper presentation at 19th EURETINA meeting, 2019, Paris.

## Results

### The characteristics of DME patients lost to follow-up

**Figure 1:**
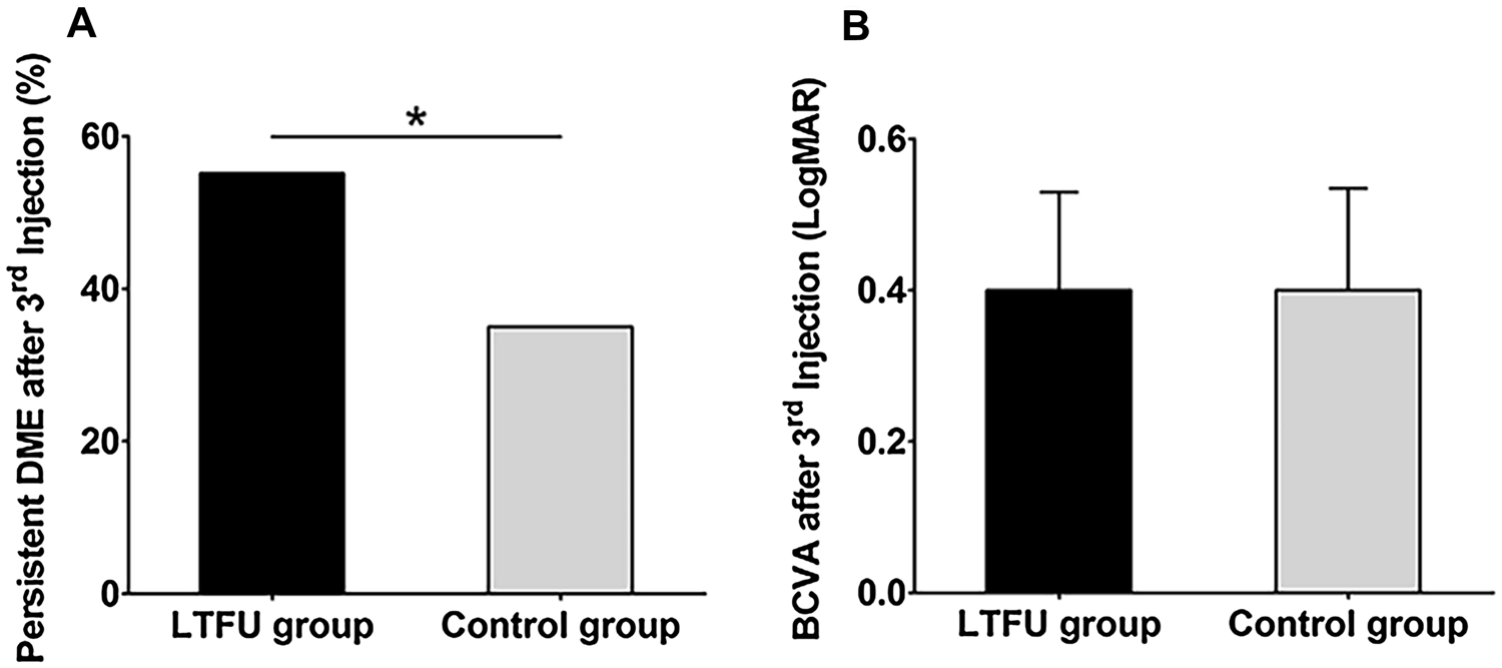
Characteristics of the DME patients lost to follow-up. (**A**) In the LTFU group, the ratio of patients with persistent macular edema is higher than in the control group (55.1% vs. 35.0%, *p* = 0.01, Chi-square test). (**B**) Between the LTFU group and control group, there is no significant difference in BCVA after the first three consecutive anti-VEGF injections.

We included 182 treatment-naïve DME patients (212 eyes). Table 1 shows the demographics of the included patients. Sixty patients (69 eyes, 33.0%) were LTFU for more than a year (LTFU group), and 122 patients (143 eyes) regularly visited the clinic during the study period (control group). The proportion of male in the LTFU group was significantly higher than that in the control group (71.67% vs. 49.18%, *p* = 0.004). The proportion of DM patients diagnosed within 5 years was significantly higher in the LTFU group than that in the control group (28.33% vs. 13.11%, *p* = 0.007). Age, duration of diabetes mellitus, hemoglobin A1c level, and best-corrected visual acuity at baseline and last visit before LTFU were not different between the two groups. Moreover, the distance from home to hospital was not different between the two groups (14.53 ± 19.89 miles vs. 12.22 ± 22.93 miles, *p* = 0.45). Figure 1 shows the visual/anatomical outcome of the LTFU and control group after three consecutive anti-VEGF injections. After the first three consecutive anti-VEGF injections, the rate of persistent DME was significantly high in the LTFU group than in the control group. Thirty-eight (55.1%) and fifty eyes (35.0%) had a foveal involving DME in the LTFU and control groups (p = 0.01, Chi-square test), respectively**.**

**Table 1:** Clinical characteristic of diabetic macular edema patients lost to follow-up for more than 1 year.

	LTFU group	Control group	*P*-value
Number	60 (33.0%)	122 (67.0%)	
Age (years)	57.80 ± 12.28	56.73 ± 12.28	0.581*
Sex (M/F)	43/17	60/62	0.004^#^
Duration of DM (years)	13.23 ± 10.19	12.81 ± 6.78	0.797*
DM duration ≤ 5 years (%)	28.33	13.11	0.007^#^
HbA1c (%)	8.40 ± 1.92	8.19 ± 2.02	0.698*
**Diabetic retinopathy grading**			
PDR	32 (53.3%)	61 (50%)	
NPDR	28 (46.7%)	61 (50%)	
Distance from home to hospital (miles)	14.53 ± 19.89	12.22 ± 22.93	0.45*
Total Follow-up (months)	11.41 ± 14.13	19.80 ± 12.45	< 0.001*
Total Anti-VEGF injection (n)	3.68 ± 2.31	5.57 ± 2.86	< 0.001*
Anti-VEGF injection at last visit before LTFU (%)	17 (28.3%)		
Baseline LogMAR BCVA	0.58 ± 0.36	0.62 ± 0.38	0.415*
LogMAR BCVA after 3rd anti-VEGF injection	0.40 ± 0.26	0.40 ± 0.27	0.895*
Baseline CST (μm)	506.32 ± 165.03	459.41 ± 134.73	0.032*
ERM at baseline (eyes)	2	2	0.452^#^
SRF at baseline (eyes)	7	21	0.360^#^
CST after 3rd anti-VEGF injection (μm)	379.84 ± 142.51	341.80 ± 94.13	0.038*

LTFU; lost to follow-up, DM; diabetes mellitus, VEGF; vascular endothelial growth factor, PDR; proliferative diabetic retinopathy, NPDR; non-proliferative diabetic retinopathy, CST; central subfield thickness, ERM; epiretinal membrane, SRF; subretinal fluid.

*Independent t-test after testing for normality using Shapiro–Wilk test.

^#^Chi-squared test.

### Visual and anatomical prognosis of the lost to follow up

Among the 60 LTFU patients, 18 patients (20 eyes, 30.0% of LTFU group) returned after loss to follow-up for more than 1 year and were re-treated with intravitreal bevacizumab injection. Table 2 presents their demographic characteristics (return group). The mean period of loss to follow-up in the return group was 23.10 ± 8.84 months. At the return visit, all twenty eyes in the LTFU patients for more than 1 year had a foveal involving DME and underwent re-treatment with bevacizumab injection.

**Table 2:** Clinical characteristics of return patients after more than 1-year loss to follow-up.

Return group	18 patients (20 eyes)
Age (years)	60.20 ± 10.09
Sex (male/female)	7/12
Type of diabetes (type I/type II)	0/19
Duration of DM (years)	12.15 ± 6.80
DR status (PDR/NPDR)	6/12
Presence of DME	20/20
HbA1c at baseline (%)	7.52 ± 1.20
HbA1c at return visit (%)	7.59 ± 0.89
Total follow-up (month)	38.78 ± 15.85
Follow-up before LTFU (month)	8.33 ± 11.20
LTFU period (month)	23.10 ± 8.84
Follow-up after re-treatment (month)	7.00 ± 5.35

LTFU; lost to follow-up.

**Figure 2:**
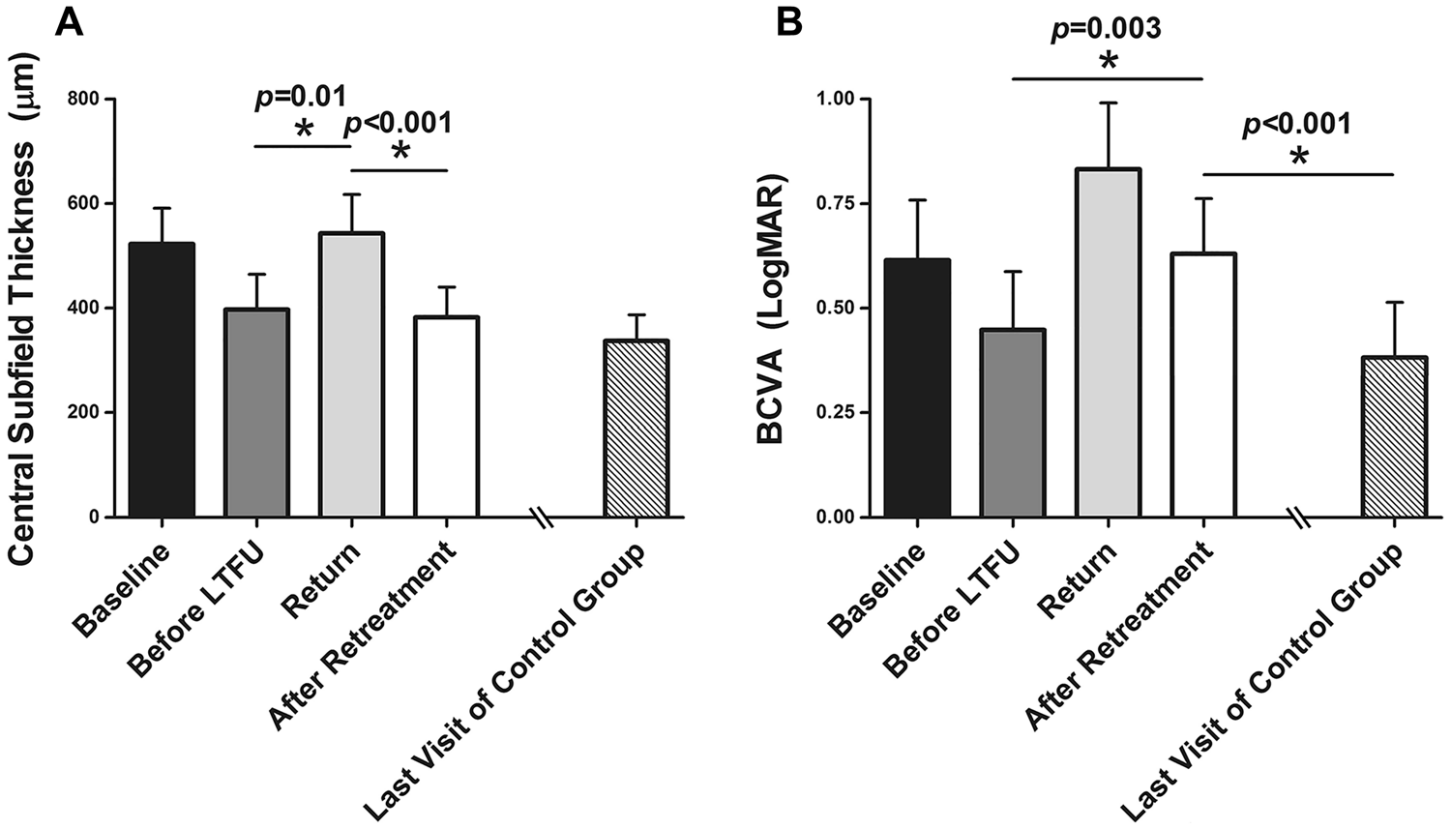
Visual and anatomical prognosis of DME patients lost to follow-up. (**A**) Central subfield thickness (CST) change in DME patients with loss to follow-up (LTFU). At the return visit, the CST was worse than the CST before LTFU. After re-treatment, the CST recovered to the level before LTFU. The CST after re-treatment in the return group was not different from the CST at the last visit of the control group. (**B**) The BCVA of DME patients with LTFU. At the return visit, the BCVA was worse than the BCVA before LTFU. After re-treatment, the BCVA was improved but did not recover to the level before LTFU. Moreover, when compared to the last visit of the control group, the BCVA after re-treatment in the return group was significantly worse.

Figure 2 shows the CST and BCVA changes in the return group. The mean CST before LTFU, upon return, and after re-treatment was significantly different (*p* < 0.05, repeated measures ANOVA). That is, the CST at return was worse than the CST before LTFU, and it improved after re-treatment. The CST at the return visit after more than 1 year of LTFU was significantly worse than that at the last visit before the loss to follow-up (542.65 ± 149.12 μm vs. 397.19 ± 134.59 μm, *p* = 0.001). After re-treatment, the CST recovered to the level before the loss to follow-up (382.11 ± 115.04 μm vs. 397.19 ± 134.59 μm, *p* = 0.47). Also, the CST after re-treatment in the return group was not significantly different than that of the control group (382.11 ± 115.04 μm vs. 336.93 ± 99.15 μm, *p* = 0.073).

**Figure 3:**
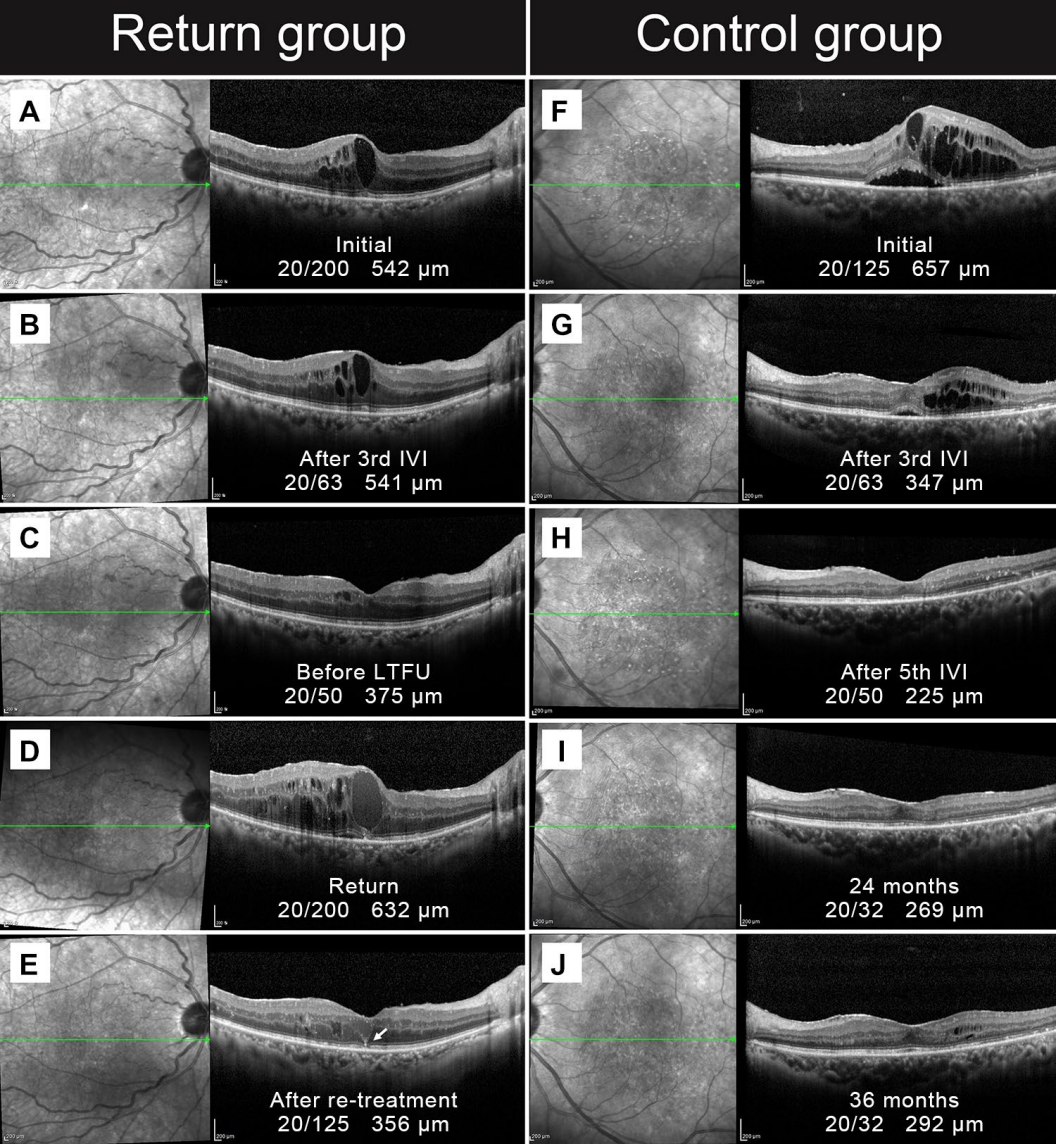
Representative cases of DME patients lost to follow-up. (**A**–**E**) A representative case of the return group. A 61-year-old man with the pseudophakic eye was lost to follow up after initial treatment with bevacizumab injection (**A**–**C**). When the patient revisited after 18 months, the BCVA and CST were worsened (**D**). After re-treatment, though the CST improved, BCVA did not recover to the previous level before LTFU. An ellipsoid zone defect (white arrow) appeared. (**F**–**J**) A representative case of the control group. A 66-year-women with pseudophakic eye regularly visited our clinic for three years. And a total of eighteen injections were administered in that period. The BCVA and CST were improved after initial treatment.

The mean BCVA before LTFU, upon return, and after re-treatment was also significantly different (*p* < 0.05, repeated measures ANOVA). That is, the BCVA at return was worse than the CST before LTFU, and it improved after re-treatment. The BCVA at the return visit after more than 1 year of LTFU was significantly worse than that before the loss to follow-up (0.83 ± 0.32 vs. 0.45 ± 0.28, *p* < 0.001). However, unlike the CST changes of the return group, BCVA did not improve to the level at the last visit before LTFU (0.63 ± 0.26 vs. 0.45 ± 0.28, *p* = 0.003). Moreover, when compared to the BCVA at the last visit in the control group, the BCVA after re-treatment in the return group was significantly worse (0.63 ± 0.26 vs. 0.38 ± 0.26, *p* < 0.001). Figure 3 shows representative cases of return and control group patients.

We analyzed the difference in ellipsoid zone defect between the return group and the control group. At the last visit before LTFU, the percentage of eyes with ellipsoid zone defect was not different between the return group and control group (Return group, 20.0% vs. Control group, 19.6%, *p* = 0.95). However, the percentage of eyes with ellipsoid zone defect in the return group significantly increased even after anti-VEGF re-treatment, while the percentage of eyes with ellipsoid zone defect in the control group who regularly visit clinic was not changed (Return group, 70.0% vs. Control group, 19.6%, *p* < 0.005). Also, the length of the ellipsoid zone defect was significantly longer in the return group than in the control group at the last visit (return group 598.72 ± 554.40, control group 145.45 ± 430.62, *p* < 0.001). In the return group, the length of the ellipsoid zone defect was significantly enlarged after re-treatment than that before LTFU (after re-treatment 598.72 ± 554.40, before LTFU 187.24 ± 437.51, *p* < 0.05, Wilcoxon signed-rank test).

### Predictive factors for lost to follow up

In multivariate regression analysis, LTFU was associated significantly with male sex (*p* = 0.004.; OR, 3.312; 95% CI, 1.474–7.444), duration of DM less than 5 years (*p* = 0.015; OR, 2.867; 95% CI, 1.226–6.707), persistent DME (*p* = 0.012; OR, 2.615; 95% CI, 1.232–5.550) (Table 3).

**Table 3:** Univariate and multivariate regression analysis of clinical characteristics associated with lost to follow-up.

Factors	Univariate		Multivariate	
	OR (95% CI)	*P*-value	OR (95% CI)	*P*-value
Sex (male)	2.614 (1.345–5.079)	0.004	3.312 (1.474–7.444)	0.004
DR grading at baseline	0.942 (0.529–1.679)	0.840		
DM duration ≤ 5 years	2.948 (1.329–6.538)	0.007	2.867 (1.226–6.707)	0.015
Persistent DME	2.280 (1.269–4.096)	0.005	2.615 (1.232–5.550)	0.012
ERM at baseline	2.104 (0.290–15.264)	0.452		
SRF at baseline	0.656 (0.264–1.627)	0.360		

DR; diabetic retinopathy, DM; diabetes mellitus, DME; diabetic macular edema, ERM; epiretinal membrane, SRF; subretinal fluid.

## Discussion

In our study, we found that 33% of DME patients were LTFU for more than 1 year during anti-VEGF treatment. When the return group received re-treatment with anti-VEGF, they achieved a significant CST improvement. However, even after re-treatment, the percentage of ellipsoid zone defect was significantly increased, and visual acuity did not recover to the previous level before LTFU.

The treatment adherence in some diabetic patients was poor and up to 50% of diabetic patients had a non-adherence to the treatment^19,20^. This poor adherence to diabetes medication is strongly associated with poor glycemic control and a higher risk for micro-and macrovascular complications of diabetes^21,22^. In DME patients particularly, poor treatment adherence might lead to LTFU during anti-VEGF treatment. In the previous study, the rate of LTFU in DME patients was higher than that in age-related macular degeneration patients^29^. We speculated that this high LTFU rate in DME patients could be the cause of the relatively poor visual outcome in real-world clinical practice, compared to the visual outcomes in clinical trials^17,18^. However, little is known about how the LTFU of DME patients during anti-VEGF treatment affects the visual/anatomical prognosis. Therefore, in this study, we evaluated the characteristics and prognosis of DME patients with LTFU.

In our study, the rate of male DME patients was significantly high in the LTFU group. One potential explanation is the difference in a relative amount of free time and employment. According to data from the National Statistical Office of our country from 2013 to 2017, the average male ratio of our city is 49.8%. However, during the same period in our city, the average employment rate of men and women was 71.4% and 52.3%, respectively. If the busy work environment affected the patient’s outpatient visit on weekdays, that would be one explanation for the male predominance in the LTFU group. There is no clear understanding as to why a higher rate of LTFU was observed in patients with diabetes mellitus less than 5 years in our study. One possible explanation is patients who have recently been diagnosed with diabetes have a poor understanding of diabetes treatment and complications, so it can be considered that the compliance to ophthalmic treatment is low. Further research is needed on whether the patient is well aware of the treatment and complications of diabetes at the time of initiating ophthalmic treatment^33^. Patients with persistent edema after three consecutive injections had poor adherence to treatment. We thought that patients with persistent edema might have poor visual acuity improvement, which makes them think that there will be no further improvement even with additional treatment. This might eventually result in poor adherence of poor early anatomical responders.

We investigated the BCVA and CST at baseline, the last visit before LTFU, the return visit, and after re-treatment in the return group patients, who re-visited the clinic after more than 1-year LTFU. When the LTFU DME patients returned to the clinic, the CST was significantly worse than that at the last visit before their LTFU. However, with anti-VEGF re-treatment in LTFU patients, the CST significantly improved to the previous level before LTFU, and CST after re-treatment was not different from that of DME patients with regular follow-up. Therefore, we could know that even if appropriate treatment is not performed for more than 1 year in LTFU patients, with the anti-VEGF re-treatment, good CST improvement could be achieved to that comparable to DME patients with regular follow-up.

However, the visual outcome in the LTFU group was different from the anatomical outcome. BCVA also became significantly worse when LTFU DME patients re-visited the clinic. However, unlike CST improvement after anti-VEGF re-treatment in LTFU patients, the BCVA did not recover to the level of the visit before LTFU. From this study, we could note the following effects of more than 1-year LTFU in the visual outcome of DME patients: (1) After more than 1 year of LTFU, the BCVA became significantly worse; (2) After re-treatment, BCVA improved significantly but not to its previous level at the visit before LTFU; and (3) the BCVA of the LTFU group after re-treatment was also significantly worse than that of the control group, who regularly visited the clinic. Considering these poor visual prognoses and increased ellipsoid zone defect in the return group, we could conclude that LTFU for more than 1 year could lead to permanent neuroretinal sensory damage in DME patients, which eventually could lead to the poor visual prognosis of LTFU DME patients. Previous clinical trials on anti-VEGF treatment in DME patients showed that delayed anti-VEGF treatment in DME patients could lead to poor visual outcomes^34^. In that study, after a 2-year anti-VEGF treatment delay in DME patients, though the anatomical improvement could be achieved, BCVA did not improve in the early continuous anti-VEGF treatment group^34^. This clinical trial result is consistent with the current study result on the poor visual outcomes of LTFU DME patients who were not properly treated for more than 1 year. And as well as delays in initial anti-VEGF treatment, discontinuation of anti-VEGF injection during treatment can also lead to significant vision loss. Furthermore, this poor visual outcome of LTFU DME patients could explain the possible reason why the visual outcome in real-world clinical practice might be worse than that in clinical trial results.

Chronic edema and fluid accumulation for long periods lead to neural cell loss over time^34^. And increased levels of serum vascular endothelial growth factor and intercellular adhesion molecule-1 levels in diabetic retinopathy are associated with an increase in the severity of diabetic retinopathy and ellipsoid zone disruption^35^. Our study showed that the length of the ellipsoid zone defect was significantly longer in the return group than in the control group at the last visit. Also, in the return group, both percentage and length of ellipsoid zone defect after retreatment were worse than that before LTFU. From these results, we could know that long-term LTFU in DME patients resulted in the permanent loss of the outer retina which was represented by an ellipsoid zone defect. And this outer retina cell loss eventually could lead to the poor visual outcomes of LTFU DME patients who were not properly treated for more than 1 year.

Therefore, considering the results of our study, if the initial treatment response is poor in a recently diagnosed diabetic patient, we should be interested in the treatment adherence and further try to increase the treatment adherence in those DME patients. Furthermore, considering the low treatment adherence in DME patients and poor visual prognosis in DME patients with LTFU, improving the patients' adherence will eventually help to improve the visual outcomes in real-world clinical practice.

The strength of the current study is that it is the first study on the prognosis and characteristics of LTFU DME patients. We found the effect of discontinuation of treatment on visual/anatomical prognosis and revealed the importance of maintaining treatment adherence in DME patients. However, the present study also had some limitations. First, this study was conducted retrospectively. As it is unethical to stop treatment for more than 1 year in DME patients for study purposes, we could not perform prospective clinical trials on the visual/anatomical outcomes in DME patients with LTFU. Therefore, a retrospective study design was an inevitable choice to confirm this effect. Second, the time interval used for the definition of LTFU was somewhat long and this may not let us capture the magnitude of the problem for the shorter LTFU periods. To know in detail about the effect of LTFU according to the duration of LTFU, we plan to conduct a future study with more participants separating the patients who lost to follow-up beyond 3 months and less than 1 year into another group. Third, we analyzed the visual/anatomical outcomes of LTFU patients using the anti-VEGF re-treatment results of return patients who LTFU for more than 1-year. In the DME patients with more than 1-year LTFU who revisited the retina clinic, a selection bias could exist since patients with a poor DME status would tend to revisit and be included in the return group. This selection bias could lead the poor visual outcome of LTFU patients. However, it is difficult to analyze the visual/anatomical prognosis of LTFU patients without this inevitable selection bias. Fourth, baseline CST in the LTFU group was significantly thicker than in the control group. it may possibly be a confounding factor. Therefore, we additionally analyzed other baseline OCT biomarkers, like subretinal fluid and epiretinal membrane, and performed binary regression analysis. We found that there was no difference in these OCT biomarkers between the two groups. Knowing changes in macular perfusion status after LTFU could give additional information about the effect of long-term LTFU in DME patients. However, we could not evaluate the changes in macular perfusion status in this current study because fluorescein angiography or OCT angiography exam could not be performed on all patients. Fifth, it would be helpful to know whether the patients have other systemic comorbidities or socioeconomic status that can be significant factors for poor adherence to ocular treatment. In a further study, it will be necessary to analyze this by using a questionnaire or telephone. Finally, although this study was conducted on a relatively large number of treatment-naïve DME patients, the number of return patients was relatively small. Therefore, further studies with more return patients might be needed to verify our study results.

In conclusion, about 30% of DME patients were lost to follow-up for more than 1 year. The male sex, DM duration within 5 years, and poor early anatomical response were key risk factors of loss to follow-up in patients with DME during anti-VEGF treatment. Also, after re-treatment, we could get a favorable CST improvement in LTFU patients. However, the ellipsoid zone defect was significantly increased and BCVA did not fully recover to the previous level of the last visit before LTFU. Therefore, considering the poor treatment adherence of DME patients, we should encourage DME patients to visit the clinic regularly. Also, patients with a high risk of LTFU such as male, early DM, and poor response after initial injections should be treated more aggressively to improve the visual outcomes.
